# It Was Trial by Fire: Recommendations for Building a Stronger Nursing Home Workforce After COVID-19

**DOI:** 10.1093/geroni/igab046.958

**Published:** 2021-12-17

**Authors:** Emily Franzosa, Wingyun Mak, Orah Burack, Kenneth Boockvar, Joann Reinhardt

**Affiliations:** 1 Icahn School of Medicine at Mount Sinai, Icahn School of Medicine at Mount Sinai, New York, United States; 2 The New Jewish Home, New York, New York, United States

## Abstract

The COVID-19 crisis showed the urgent need for a unified, well-supported nursing home workforce. The objective of this qualitative study was to examine the lived experience of certified nursing assistants (CNAs) and administrators during COVID-19 to identify best practices moving forward. Six administrator interviews and 10 remote focus groups with CNAs at 5 nursing homes (N=56) were examined through directed content analysis. Based on priorities identified by CNAs and administrators, the following practices may be most impactful: 1) ongoing and responsive staff training; 2) transparent, direct, and two-way communication channels; 3) prioritizing hiring permanent staff to avoid shortages and reliance on agency staff; 4) building collaborative staff-management relationships; 5) providing flexible job benefits; 6) providing staff-centered emotional support resources; and 7) appraising COVID-19 innovations. Our results suggest that rather than returning to “business as usual,” nursing homes can draw on these lessons to build a more sustainable workforce and industry.

